# Golimumab for Rheumatoid Arthritis

**DOI:** 10.3390/jcm8030387

**Published:** 2019-03-20

**Authors:** Eleftherios Pelechas, Paraskevi V. Voulgari, Alexandros A. Drosos

**Affiliations:** Rheumatology Clinic, Department of Internal Medicine, Medical School, University of Ioannina, 45110 Ioannina, Greece; pelechas@doctors.org.uk (E.P.); pvoulgar@cc.uoi.gr (P.V.V.)

**Keywords:** rheumatoid arthritis, TNFα, golimumab, efficacy, tolerability, immunogenicity

## Abstract

Since the advent of infliximab for the treatment of rheumatoid arthritis (RA), new genetically-engineered molecules have appeared. This review aims to present the current data and body of evidence for golimumab (GLM). Safety, efficacy, tolerability and immunogenicity are all being investigated, not only through phase III trials (GO-BEFORE, GO-FORWARD, GO-AFTER, GO-MORE, GO-FURTHER, GO-NICE), but also through studies of real-world data. It seems that GLM in the subcutaneous form is an efficacious molecule with a good safety profile at the standard dosage scheme, but a 100 mg subcutaneous dose is associated with a higher risk of opportunistic infections, lymphoma and demyelination. Furthermore, when compared to other tumor necrosis factor-α molecules, it is non-inferior, and, at some points, such as when it comes to immunogenicity and persistence of the drug, it has a better profile. In summary, GLM is an effective, well-tolerated option for the treatment of RA, for both the clinician and patients who are seeking a convenient dosage scheme.

## 1. Introduction

Nowadays, rheumatology has been transformed into one of the most impactful specialties in the field of medicine, mainly due to a better understanding of the way our immune system responds to different internal and external stimuli [1]. The idea of neutralizing tumor necrosis factor (TNF)α via a specific antibody emerged in the mid-1980s. The hypothesis was that reducing TNFα levels would restore the balance in the cytokine system. Thus infliximab (INF), a chimeric human-murine monoclonal antibody that binds with high affinity to both soluble and transmembrane forms of TNFα, but not to lymphotoxin α (TNFβ), was developed with the employment of genetic engineering techniques. Since the advent of INF, four more genetically engineered molecules have been marketed: etanercept (ETN), adalimumab (ADA), certolizumab (CTZ) and golimumab (GLM), each employing a slightly different compositional and pharmacodynamic approach. In addition, anti-TNFα biosimilars have come of age and are already on the market [2,3]. Nevertheless, even with the appearance of different molecules targeting rheumatoid arthritis (RA), the unmet needs for the treatment of the disease remain high [4].

## 2. Golimumab

GLM is a human IgG1κ monoclonal antibody produced by a murine hybridoma cell line with recombinant DNA technology [5], which has been shown to improve the signs and symptoms of RA in adults in large, randomized, placebo-controlled phase III trials [6,7,8,9,10]. It is the latest anti-TNFα approved by the Food and Drug Administration (FDA), in 2009, under the brand name Simponi. In Europe, a once-monthly 50-mg subcutaneous (s.c.) formulation of the TNFα GLM is approved as monotherapy and/or in combination with methotrexate (MTX). Other approved indications of GLM are for the treatment of psoriatic arthritis (PsA) and axial spondyloarthritis (AxSpA)—comprising ankylosing spondylitis (AS) and non-radiographic axial spondyloarthritis (nr-AxSpA) in adults, and polyarticular juvenile idiopathic arthritis (pJIA) in children (50 mg/month if body weight >40 kg). In patients with body weight greater than 100 kg and for all the above indications who do not achieve an adequate clinical response after 3–4 doses, increasing the dose to 100 mg once a month may be considered, taking into account the increased risk of certain serious adverse drug reactions. Finally, GLM has been also approved for ulcerative colitis. The initial dose should be 200 mg, followed by 100 mg at week two. Patients who have an adequate response should receive 50 mg at week six and every four weeks thereafter, whereas for those with an inadequate response or with body weight greater than 80 kg, 100 mg at week six and every four weeks thereafter.

## 3. Pharmacological Properties of GLM

GLM acts principally by targeting and neutralizing TNFα with the ultimate goal to prevent inflammation as well as cartilage degradation and bone destruction [11]. In pivotal phase III trials (but also in different sub-studies in patients with RA and other inflammatory arthritides), when administered alone or in combination with MTX, it showed that there is a significant reduction in serum acute phase reactants and other inflammatory biomarkers [12,13,14,15].

GLM exhibits dose-proportional pharmacokinetics and this is why patients with different body weights should receive different dosage schemes. The median time to maximum plasma concentration is 2–7 days following a single s.c. injection. Steady-state plasma concentrations can be achieved at 12 weeks of repeated injections and the mean absolute bioavailability is approximately 50% [16]. The mean elimination half-life is estimated to be approximately 12 days. In patients receiving MTX with GLM, the mean steady-state trough concentrations were 30% higher than those receiving GLM alone. The concomitant use of MTX reduces the apparent clearance of GLM by approximately 35% [17].

## 4. Clinical Efficacy

The clinical efficacy of GLM in inflammatory arthritides has been shown in a series of phase III trials but also in several sub-studies (Table 1) [8,9,10,18,19,20,21,22,23]. More specifically, in RA there is sufficient data supporting the therapeutic efficacy of the drug.

In the GO-BEFORE study (NCT00264537), a total of 637 MTX-naïve patients with active RA were randomized (1:1:1:1) to placebo + MTX (group 1), GLM 100 mg + placebo (group 2), GLM 50 mg + MTX (group 3), or GLM 100 mg + MTX (group 4). This study did not detect significant differences in ACR50 response (primary endpoint) between the combination therapy groups (3 and 4) of GLM (50 mg/100 mg) every four weeks plus MTX and MTX as monotherapy. A difference would have been seen if the ACR 20 response had been considered. Thus, the modified intend-to-treat (ITT) analysis of the primary endpoint and other prespecified efficacy measures demonstrated that the efficacy of GLM + MTX is better than, and the efficacy of GLM alone is similar to, the efficacy of MTX alone in reducing RA symptoms in MTX naïve patients, with no unexpected safety concerns [8,18].

In the *GO-FORWARD* study (NCT00264550), a total of 444 patients with active RA despite MTX therapy were randomly assigned (3:3:2:2) to placebo injections + MTX capsules (group 1), GLM 100 mg injections + placebo capsules (group 2) GLM 50 mg injections + MTX capsules (group 3) and GLM 100 mg injections + MTX capsules (group 4). The co-primary endpoints were the proportion of patients with >ACR20% improvement at week 14 and change from baseline in the health assessment questionnaire-disability index (HAQ-DI) score at week 24. In the aforementioned groups ACR20 response at week 14 was achieved by 33.1%/44.4%/55.1%/56.2%, respectively, whereas at week 24, median improvements from baseline in HAQ-DI score (0.13) were: 0.13 (*p* = 0.240); 0.38 (*p* < 0.001); 0.50 (*p* < 0.001), respectively [9,19]. The conclusion of this study was that the addition of GLM to MTX in patients with active RA despite MTX therapy, significantly reduced the signs and symptoms of RA and improvement of physical function.

The *GO-AFTER* study (NCT00299546) evaluated the efficacy and safety of GLM in subjects who have active RA and have been treated previously with >1 dose of a biologic anti-TNFα agent (ETN, ADA, INF). A total of 461 patients from 10 countries were randomly allocated to receive s.c. injections of placebo (group 1), GLM 50 mg s.c. (group 2) or GLM 100 mg s.c. (group 3) every four weeks. MTX, sulfasalazine (SSZ), hydroxychloroquine (HCQ), oral corticosteroids (CS) and non-steroidal anti-inflammatory drugs (NSAIDs) were carried on at stable doses. As primary endpoint, an ACR20 improvement at week 14 should be achieved by patients who discontinued previous anti-TNFα treatment due to lack of effectiveness or reasons unrelated to effectiveness, such as intolerance and accessibility issues. In groups 1–3, 18%/35%/38% respectively achieved ACR 20 at week 14. The conclusion of this study was that GLM reduces the signs and symptoms of RA in patients with active disease who had previously received >1 anti-TNFα [10,20].

In the *GO-MORE* study (NCT00975130) a total number of 3366 patients were enrolled in order to evaluate the efficacy and safety of s.c. GLM as add-on therapy in patients with active RA in typical clinical practice settings (use of csDMARDs and Cs). A four-weeks add-on of 50 mg s.c. GLM for a period of six months were given in part one of the study whereas in part two, patients not on remission were randomly assigned to receive intravenous (i.v.) + s.c. (group 1) or s.c. GLM to month 12. Neither in part one nor part two of the study a statistically significant difference was observed apart from the efficacy and safety of GLM as an add-on therapy for csDMARD-refractory RA in a typical clinical practice population. This study concluded that there is no additional efficacy of the i.v. + s.c. scheme of GLM over the s.c. regimen [21].

The *GO-FURTHER* study (NCT00973479) evaluated not only the safety and efficacy but also the radiographic progression through two years of treatment with i.v. GLM + MTX in an open-label extension of a phase III trial of patients with active RA despite MTX therapy. A total number of 592 patients with active RA were randomized (2:1) to i.v. GLM 2 mg/kg + MTX (group one), or placebo + MTX (group 2) at weeks 0 and 4, and every eight weeks thereafter. ACR 20/50/70 response criteria were measured as well as the 28-joint count disease activity score using the C-reactive protein (DAS-28-CRP), physical function and quality of life, and changes in the modified Sharp/van der Heijde scores (SHS). The ACR responses at week 100 were 68.1%/43.8%/23.5% respectively. Physical function, quality of life and clinical response were maintained throughout the study period (two years). The SHS score was 0.74 in group 1 and 2.10 in group 2 (*p* = 0.005). As far as it concerns the AE, 79.1% had at least one and 18.2% had a serious AE. This study demonstrated that in patients with active RA, despite MTX, i.v. GLM + MTX showed significant inhibition of structural damage at weeks 24 and 52 and substantial clinical improvement with no safety signs up to one year [22].

The *GO-NICE* study (NCT01313858), aimed to document patient and treatment characteristics as well as clinical effectiveness and safety in adult patients newly treated with the 50 mg s.c. GLM every four weeks under real-life conditions. Of the 1613 patients, 1458 were eligible for final analysis and of those 474 patients were suffering from RA. The mean age of those patients was 54.9 ± 13.4 years, 72.8% were females and 64.7% biologic-naïve. The DAS-28-erythrocyte sedimentation rate (ESR) decreased from 5.0 to 2.9 after 24 months (*p* < 0.0001). As reported, most AE were of mild or moderate nature, and no new safety signals were detected [23].

Finally, there are several other studies regarding the persistence of GLM treatment in patients with RA. Thomas et al. [24] in a retrospective, observational study of all patients treated with GLM in four Academic centers in Greece during a four-year period examined the long-term survival on drug (SOD) of patients not only with RA (166 patients) but also PsA (82 patients) and AS (80 patients). The estimated SOD at two and three years was 68% and 62% respectively (69% and 60% for RA patients) concluding that GLM showed a high three-year SOD with a low rate of discontinuation due to AEs. Furthermore, Rotar et al. [25] analyzed prospectively the collected data of all patients treated with GLM and other TNFs for seven years and were suffering from RA, AS, and PsA. The authors concluded that the persistence of GLM in RA-treated patients is lower compared with the AS and PsA patients but it is higher among those patients treated with other anti-TNFs. Svedbom et al. [26] in a systematic review of real-world evidence in immune-mediated rheumatic diseases including RA, examined the persistence to treatment with s.c. GLM but also to other anti-TNFα molecules. Of 376 available references identified, 12 studies with a total of 4910 patients met the inclusion criteria. In four studies that included comparisons to other biologics, GLM was either statistically noninferior or statistically superior to other treatments. Serrano et al. [27] in a prospective monocentric cohort of RA patients treated with GLM and a total number of 61 patients (mean age 55.1 ± 14.1 years; 85.2% females; RF + 70%; anti-CCP + 78%) showed that GLM survival time was better when used as first or second biological and with concomitant use of csDMARDs. Aaltonen et al., based on Kaplan-Meier survival analysis in a systematic review regarding the anti-TNFα, showed that the probability of discontinuing the treatment within 6, 12, 24, and 36 months was 16%, 27%, 37%, and 43%, respectively in patients with RA. SOD was better among the patients with no prior bDMARD therapy than among those using anti-TNFα as their second or third bDMARD. CTZ (41%) and INF (38%) were associated with higher probability of treatment discontinuation within 12 months compared to ADA (25%), ETN (25%), and GLM (25%) [28].

## 5. Tolerability and Immunogenicity

Data from the pivotal phase III trials in adults with RA but also the open-label extension studies, support that s.c. GLM is generally a well-tolerated therapeutic option [8,9,10]. Overall, in these trials, upper-respiratory infections (32.0% vs. 8.8% with placebo), nasopharyngitis (17.4% vs. 6.4%), followed by elevated aminotransferase levels (11.9% vs. 5.2%) and hypertension (9.8% vs. 2.7%) were the most common AE in the 50 mg s.c. dose. Injection-site reactions (ISRs) were reported by 11.0% vs. 2.8% of GLM and placebo recipients, the most common being injection-site erythema (5.8% vs. 1.1%) [29]. Tuberculosis, opportunistic infections, lymphoma, and demyelination incidence appeared to be higher among patients receiving GLM 100 mg s.c. dose.

As far as it concerns the immunogenicity, Thomas et al. documented in a systematic review for the immunogenicity of TNF inhibitors that GLM and ETN were the least immunogenic (3.8% and 1.2% respectively) whereas the most immunogenic were INF (25.3%), followed by ADA (14.1%) and CTZ (6.9%). The clinical significance of the anti-drug antibodies (ADAbs) in the sera of patients with RA is associated with decreased clinical response [30].

## 6. Conclusions

GLM is one of five anti-TNFα inhibitors approved for the treatment of RA, but also other inflammatory arthritides [31]. It is a newer, second-generation anti-TNFα and for this reason the clinical experience is less in comparison with the older ones such as INF, ETA and ADA (first generation TNFα inhibitors). On the other hand, the growing body of evidence through the open-label extension trials of pivotal studies and those from several medical centers in patients with RA, confirm the efficacy and safety of the drug. Furthermore, with clinical and radiological benefits being sustained and no new safety signals being identified, GLM seems an attractive choice for the treatment of RA. Other, important elements that make this choice attractive, are the low levels of immunogenicity, the low rate of drug discontinuation in comparison with the other anti-TNFs and the dosage scheme (every four weeks) which seems to be a point of major significance when a physician-patient sharing decision occurs. One concern is the tendency of higher incidence of opportunistic infections, lymphoma and demyelination in the 100 mg s.c. injection, and it should be used with caution in patients with higher body weight or poor response to treatment with the 50 mg dosage scheme.

As there are no head-to-head trials comparing it with the other anti-TNFα inhibitors, the indirect comparison of all five agents suggests that possibly GLM is better tolerated than ADA, CTZ and INF in terms of the risks of serious infection and of discontinuing treatment due to AEs. In summary, GLM is an effective, well-tolerated option for the treatment of RA for both the clinician but also for the patients seeking a convenient dosage scheme.

## Figures and Tables

**Table 1:** Summary of GLM trials.

Trial (Clinical Trial Identifier Number)	Official Title	Study Type (Phase)	Indication	Number of Participants
GO-BEFORE (NCT00264537)	A multicentre, randomized, double-blind, placebo-controlled trial of golimumab, a fully human anti-TNFa monoclonal antibody, administered subcutaneously, in methotrexate-naïve subjects with active rheumatoid arthritis	Clinical Trial (Phase III)	RA	637
GO-FORWARD (NCT00264550)	A multicentre, randomized, double-blind, placebo-controlled trial of golimumab, a fully human anti-TNFa monoclonal antibody, administered subcutaneously, in subjects with active Rheumatoid arthritis despite methotrexate therapy	Clinical trial (Phase III)	RA	444
GO-AFTER (NCT00299546)	A multicentre, randomized, double-blind, placebo-controlled trial of golimumab, a fully human anti-TNFa monoclonal antibody, administered subcutaneously in subjects with active rheumatoid arthritis and previously treated with biologic anti-TNFa Agent(s)	Clinical trial (Phase III)	RA	461
GO-MORE (NCT00975130)	An open-label study assessing the addition of subcutaneous golimumab (GLM) to conventional disease-modifying antirheumatic drug (DMARD therapy in biologic-naïve subjects with rheumatoid arthritis (Part 1), followed by a randomized study assessing the value of combined intravenous and subcutaneous GLM administration aimed at inducing and maintaining remission (Part 2)	Clinical trial (Phase III)	RA	3366
GO-FURTHER (NCT00973479	A multicentre, randomized, double-blind, placebo-controlled trial of golimumab, an anti-TNF alpha monoclonal antibody, administered intravenously, in patients with active rheumatoid arthritis despite methotrexate therapy	Clinical trial (Phase III)	RA	592
GO-NICE (NCT01313858)	Non-interventional study investigating the use of golimumab in patients with rheumatoid arthritis, psoriatic arthritis, and ankylosing spondylitis	Observational	RA, PsA, AS	1613

GLM: golimumab; TNF: tumor necrosis fator; RA: rheumatoid arthritis; PsA: psoriatic arthritis; AS: ankylosing spondylitis; nr-AxSpA: non-radiographic axial spondyloarthritis.
